# Elevated Tumor HIF-1α Expression Correlates with Advanced Pathological Stage Following Neoadjuvant Concurrent Chemoradiotherapy in Esophageal Squamous Cell Carcinoma

**DOI:** 10.3390/cimb48050525

**Published:** 2026-05-18

**Authors:** Hsin-Yi Shih, Chien-Chih Chen

**Affiliations:** 1 Department of Radiation Oncology, Taichung Veterans General Hospital, Taichung 407219, Taiwan; 2 Department of Medical Imaging and Radiological Sciences, Central Taiwan University of Science and Technology, Taichung 406053, Taiwan

**Keywords:** HIF-1α, esophageal cancer, neoadjuvant concurrent chemoradiotherapy

## Abstract

Tumor hypoxia has been implicated in treatment resistance and disease progression in esophageal squamous cell carcinoma (ESCC), yet its relationship with post-neoadjuvant pathological staging remains unclear. This study evaluated the association between hypoxia-inducible factor-1α (HIF-1α) expression and pathological stage following neoadjuvant concurrent chemoradiotherapy (CCRT). We retrospectively analyzed 55 patients with ESCC treated with standardized neoadjuvant CCRT followed by curative esophagectomy. Immunohistochemical staining was performed on surgical specimens to assess tumor (HIF-T%) and stromal (HIF-N%) HIF-1α expression, and correlations with postoperative pathological stage were analyzed. Tumor HIF-1α expression was significantly higher in patients with pathological stage III disease compared with stage I–II disease (40% vs. 15%, *p* = 0.023). Increasing trends in tumor HIF-T% were observed across higher T and N classifications, although these did not reach statistical significance. Stromal HIF-1α expression was not associated with pathological stage. These findings demonstrate that elevated tumor HIF-1α expression is associated with advanced pathological stage following neoadjuvant CCRT in ESCC, supporting the role of hypoxia-related signaling in treatment resistance. HIF-1α may serve as a clinically relevant biomarker of residual disease burden, although further validation in larger cohorts is warranted.

## 1. Introduction

Esophageal cancer (EC) remains a major global oncologic challenge, ranking as one of the leading causes of cancer-related mortality worldwide [1,2]. Despite ongoing advances in multimodality treatment, including surgery, radiotherapy, and systemic chemotherapy, long-term survival remains unsatisfactory. Globally, EC accounts for more than 600,000 new cases annually, with mortality rates closely paralleling incidence rates [2]. Esophageal squamous cell carcinoma (ESCC) is the most common histological subtype of esophageal cancer in East Asia, including Taiwan, whereas esophageal adenocarcinoma is more common in Western populations [3]. Even with neoadjuvant concurrent chemoradiotherapy (CCRT) followed by surgical resection, the reported 5-year survival rate remains between 15 and 25% [3,4]. These outcomes underscore the need for biologically informed prognostic markers that may refine risk stratification beyond conventional staging systems.

Tumor hypoxia is widely recognized as an important biological factor contributing to tumor progression and resistance to anticancer therapies. Hypoxia-inducible factor-1 alpha (HIF-1α) is a master transcriptional regulator activated under low oxygen tension and mediates adaptive cellular responses involved in angiogenesis, metabolic reprogramming, invasion, and survival. Stabilization of HIF-1α has been documented across multiple solid malignancies and is frequently associated with aggressive clinical behavior [5,6]. Mechanistically, HIF-1α upregulates genes involved in glycolytic metabolism, vascular endothelial growth factor (VEGF) signaling, and epithelial–mesenchymal transition, thereby facilitating tumor progression and resistance to cytotoxic therapy [7,8,9].

In several cancer types, elevated HIF-1α expression has been linked to adverse outcomes. In breast and gastrointestinal malignancies, increased HIF-1α correlates with higher tumor grade, enhanced metastatic potential, and reduced survival [8,10]. Furthermore, experimental evidence demonstrates that HIF-1α contributes to chemoresistance through modulation of multidrug transporters and DNA damage response pathways [9]. These findings support the concept that hypoxia-driven signaling represents not merely a passive tumor characteristic but an active driver of therapeutic failure.

In ESCC, several studies have suggested that HIF-1α overexpression is associated with poor response to chemoradiotherapy, advanced tumor stage, and inferior survival outcomes [5,10,11,12]. These findings support the role of hypoxia-related signaling in treatment resistance. However, most existing studies have primarily focused on survival endpoints or composite biomarker panels. As a result, the quantitative relationship between tumor HIF-1α expression and post-neoadjuvant pathological staging remains insufficiently characterized. In addition, the clinical significance of HIF-1α remains inconsistent across studies, likely due to differences in study design, treatment context, and evaluation methods.

Given that pathological stage following neoadjuvant therapy remains one of the strongest determinants of prognosis in ESCC, clarifying the relationship between hypoxia signaling and residual tumor burden is clinically relevant. We therefore conducted a retrospective analysis to evaluate the association between HIF-1α expression levels in resected tumor specimens and pathological staging in ESCC patients treated with neoadjuvant CCRT. By focusing on tumor-specific expression patterns and stratified pathological outcomes, this study aims to further define the clinical significance of hypoxia-related signaling in the modern multimodality treatment era. Our findings demonstrate a significant association between tumor HIF-1α expression and advanced pathological stage following neoadjuvant therapy.

## 2. Materials and Methods

### 2.1. Patients

This retrospective cohort study included consecutive patients with newly diagnosed ESCC who underwent neoadjuvant CCRT followed by curative-intent esophagectomy at Taichung Veterans General Hospital between January 2011 and December 2015.

Eligibility criteria included (1) histologically confirmed ESCC, (2) completion of neoadjuvant CCRT, (3) availability of formalin-fixed paraffin-embedded (FFPE) tumor specimens from surgical resection, and (4) complete clinicopathologic data, including post-treatment pathological staging.

Patients with distant metastatic disease at diagnosis or incomplete treatment records were excluded. Clinical staging before neoadjuvant therapy was determined according to the 8th edition AJCC TNM classification system. Pathological staging (pT and pN classifications) was assigned based on postoperative histopathologic evaluation according to the same AJCC criteria.

The study protocol was approved by the Institutional Review Board of Taichung Veterans General Hospital (IRB protocol code CE21111A-4; date of approval: 15 April 2025). Given the retrospective nature of the study, the requirement for informed consent was waived.

### 2.2. Chemoradiotherapy

All patients received concurrent chemoradiotherapy as neoadjuvant treatment.

Radiotherapy was delivered to a total dose of 50 Gy in 25 fractions (2.0 Gy per fraction, five fractions per week) using volumetric modulated arc therapy (VMAT; RapidArc technique) with a linear accelerator operating under the source-to-axis distance (SAD) technique. Target volumes were defined according to institutional protocols, encompassing the primary tumor and regional lymphatics.

Concurrent chemotherapy consisted of cisplatin (20 mg/m^2^/day) and 5-fluorouracil (5-FU) (800 mg/m^2^/day). Two cycles were administered: days 1–4 and days 29–32 of the radiotherapy course.

### 2.3. Surgery

Surgical resection was performed 4–6 weeks after completion of neoadjuvant CCRT. All patients underwent esophagectomy with regional lymph node dissection. Gastroesophageal continuity was restored using gastric conduit reconstruction. Resection margins and lymph node specimens were examined by dedicated gastrointestinal pathologists.

### 2.4. Tissue Sample Preparation and Immunohistochemistry (IHC) Staining

FFPE tumor blocks obtained from surgical specimens were sectioned at 4 μm thickness. Sections were deparaffinized in xylene and rehydrated through graded ethanol solutions. Hematoxylin and eosin (H&E) staining of adjacent sections was performed for pathological confirmation of tumor regions prior to immunohistochemical evaluation.

Immunohistochemical staining for HIF-1α was performed using a rabbit polyclonal anti-HIF-1α antibody (Proteintech, Cat. No. 20960-1-AP). The staining protocol was standardized as follows: 1. Antigen retrieval: heat-induced epitope retrieval was conducted in citrate buffer (pH 6.0) at 98 °C for 20 min; 2. Blocking: endogenous peroxidase activity was quenched, followed by incubation in 5% bovine serum albumin for 30 min to reduce nonspecific binding; 3. Primary antibody incubation: sections were incubated overnight at 4 °C with the primary antibody at a dilution of 1:200 in a humidified chamber; 4. Secondary detection: after washing in phosphate-buffered saline (PBS), sections were incubated with a biotinylated secondary antibody, followed by streptavidin–horseradish peroxidase (HRP) detection system according to the manufacturer’s instructions; 5. Visualization: signal detection was achieved using 3,3′-diaminobenzidine (DAB), producing a brown chromogenic reaction product; 6. Counterstaining: Slides were counterstained with hematoxylin, dehydrated, and mounted.

Negative controls were processed identically except for omission of the primary antibody.

### 2.5. Scoring of HIF-1α Expression

HIF-1α expression was independently evaluated by two experienced pathologists who were blinded to clinical outcomes. Expression was assessed in both tumor cells (HIF-T) and adjacent stromal/non-tumor cells (HIF-N). Tumor regions were identified based on histopathologic features confirmed by hematoxylin and eosin (H&E) staining. Cells within these regions were classified as tumor cells, whereas surrounding non-tumor cells were classified as stromal cells for HIF-N evaluation.

**Figure A1:**
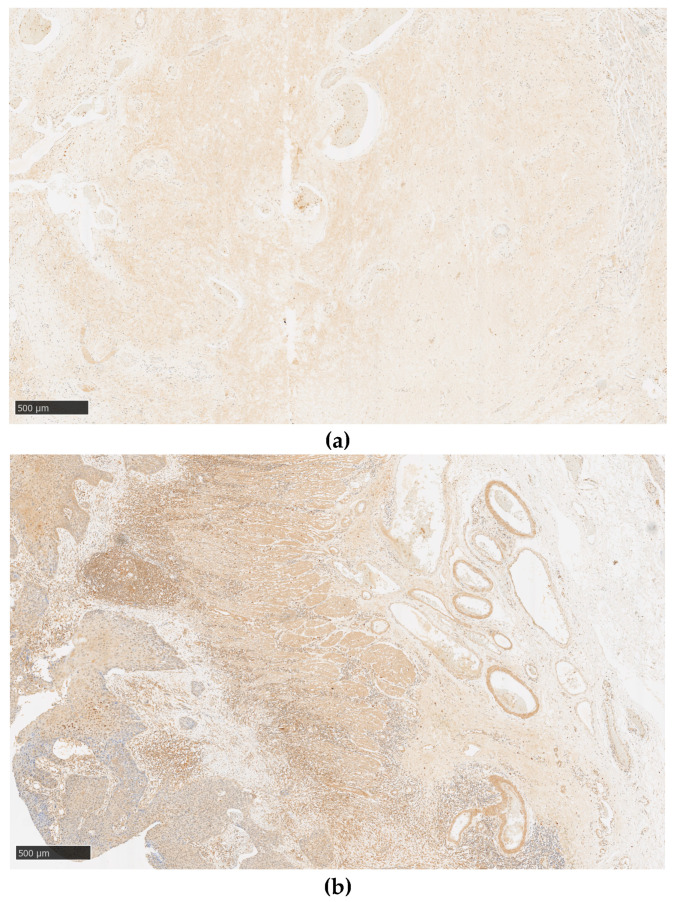

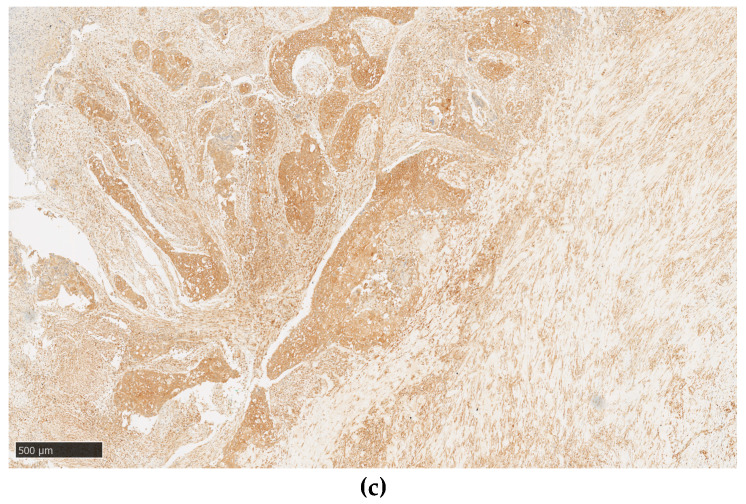
Representative immunohistochemical staining of HIF-1α in tumor cells at 4× magnification illustrating the grading criteria for staining intensity. Scale bar = 500 μm. (**a**) Grade 0 (negative) staining with no detectable nuclear or cytoplasmic immunoreactivity in tumor cells. (**b**) Grade 1 (weak) staining showing faint nuclear or cytoplasmic immunoreactivity in tumor cells. (**c**) Grade 2 (moderate) staining, demonstrating clearly visible nuclear or cytoplasmic immunoreactivity in tumor cells.

**Figure A2:**
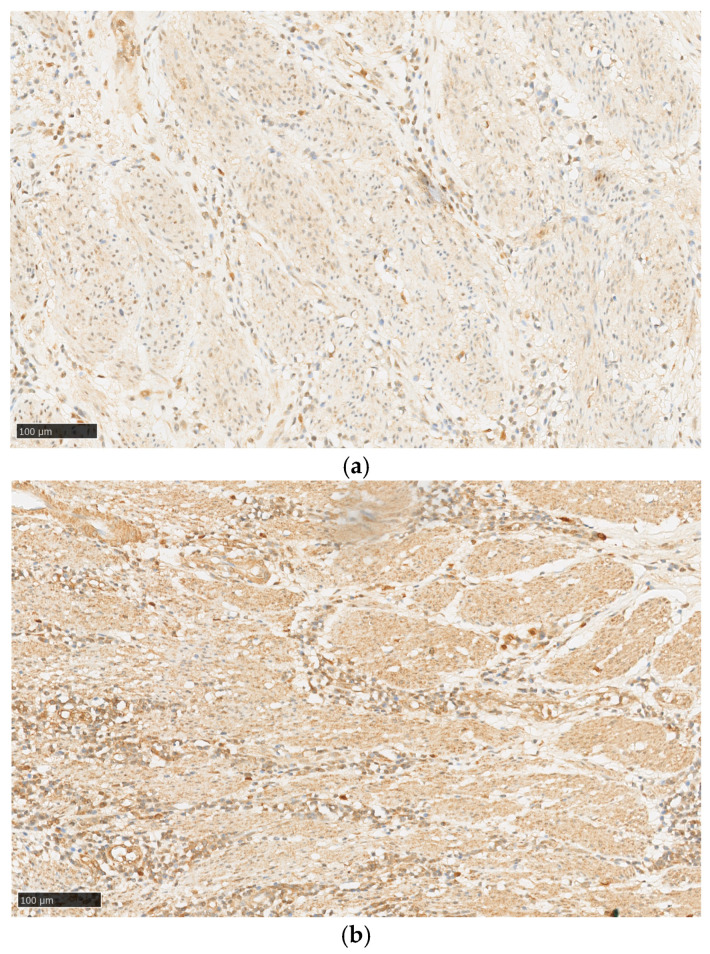

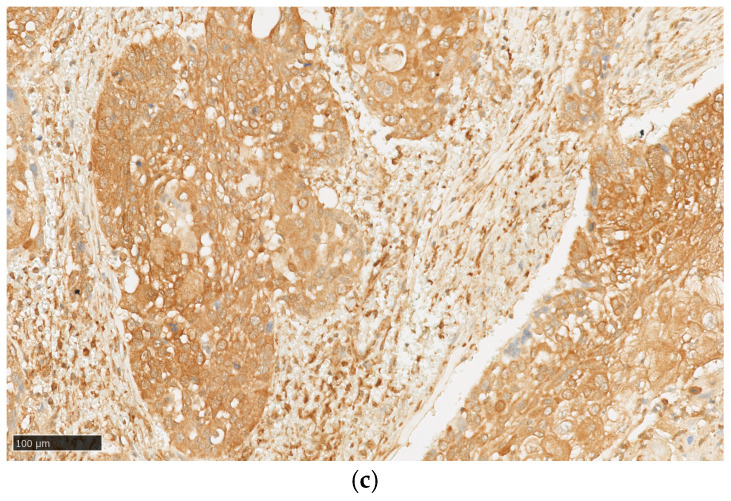
Representative immunohistochemical staining of HIF-1α in tumor cells at 20× magnification illustrating the grading criteria for staining intensity. Scale bar = 100 μm. (**a**) Grade 0 (negative) staining with no detectable nuclear or cytoplasmic immunoreactivity in tumor cells. (**b**) Grade 1 (weak) staining showing faint nuclear or cytoplasmic immunoreactivity in tumor cells. (**c**) Grade 2 (moderate) staining, demonstrating clearly visible nuclear or cytoplasmic immunoreactivity in tumor cells.

**Figure A3:**
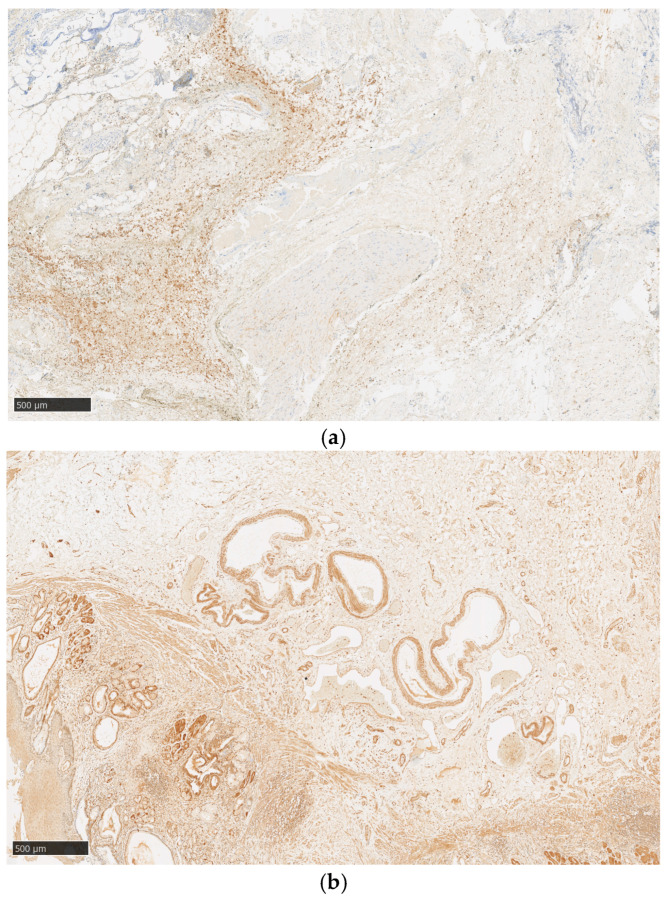

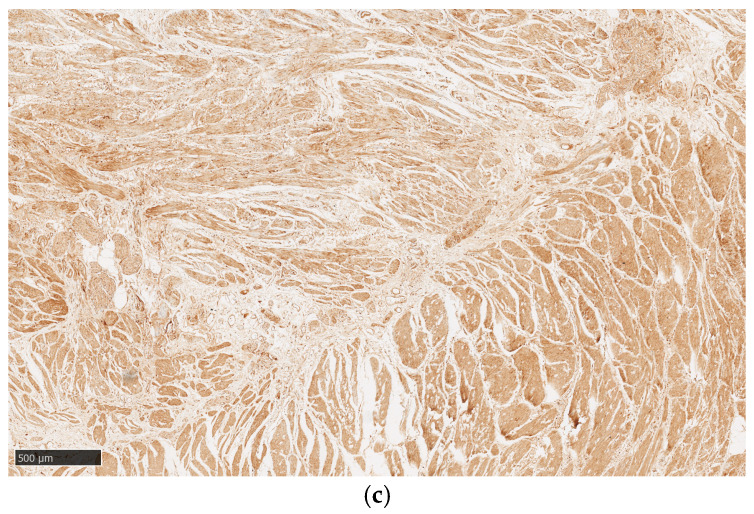
Representative immunohistochemical staining of HIF-1α in stromal cells at 4× magnification illustrating the grading criteria for staining intensity. Scale bar = 500 μm. (**a**) Grade 0 (negative) staining with no detectable nuclear or cytoplasmic immunoreactivity in stromal cells. (**b**) Grade 1 (weak) staining showing faint nuclear or cytoplasmic immunoreactivity in stromal cells. (**c**) Grade 2 (moderate) staining demonstrating clearly visible nuclear or cytoplasmic immunoreactivity in stromal cells.

**Figure A4:**
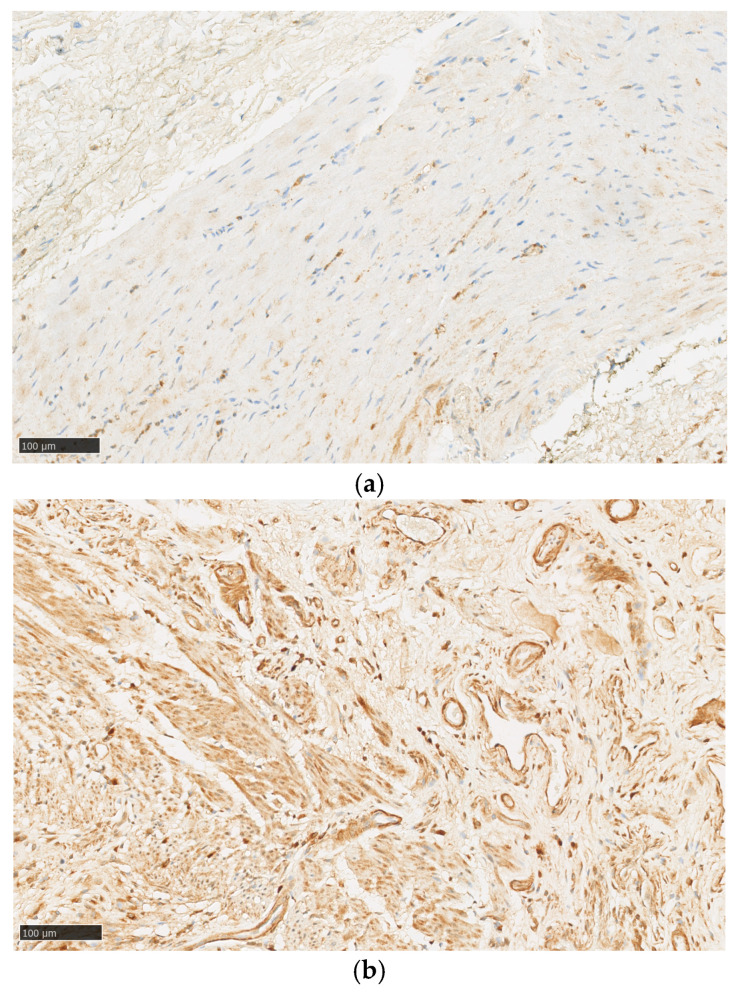

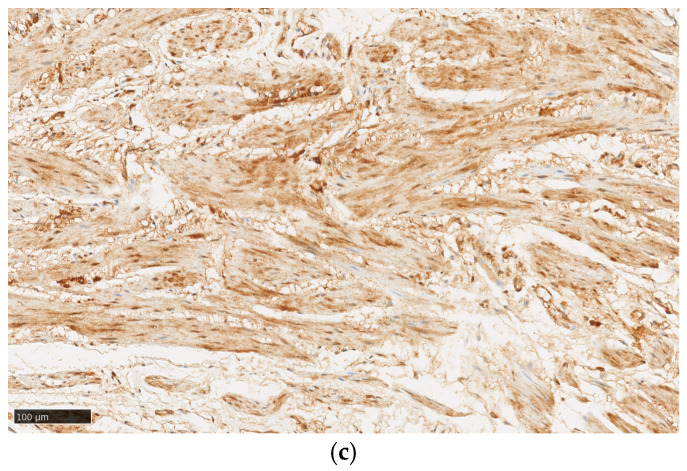
Representative immunohistochemical staining of HIF-1α in stromal cells at 20× magnification illustrating the grading criteria for staining intensity. Scale bar = 100 μm. (**a**) Grade 0 (negative) staining with no detectable nuclear or cytoplasmic immunoreactivity in stromal cells. (**b**) Grade 1 (weak) staining showing faint nuclear or cytoplasmic immunoreactivity in stromal cells. (**c**) Grade 2 (moderate) staining, demonstrating clearly visible nuclear or cytoplasmic immunoreactivity in stromal cells.

Two parameters were recorded: (1) percentage of positive cells (% HIF-T or % HIF-N), defined as the proportion of tumor or stromal/non-tumor cells demonstrating nuclear and/or cytoplasmic immunoreactivity among the total number of cells within representative high-power fields; and (2) staining intensity, graded as follows: 0 (negative), 1 (weak), 2 (moderate), and 3 (strong) (Figure A1, Figure A2, Figure A3 and Figure A4). Discrepancies between the two pathologists were resolved by joint review to reach consensus. No cases met the criteria for grade 3 staining.

### 2.6. Statistical Analysis

Statistical analyses were performed using SPSS software version 25 (IBM Corp., Armonk, NY, USA). Continuous variables were expressed as median with interquartile range (IQR) and compared using the Mann–Whitney U test. Categorical variables were compared using the chi-square test or Fisher’s exact test, as appropriate.

To further evaluate whether HIF-1α expression was independently associated with advanced pathological stage, logistic regression analysis was performed using pathological stage III disease as the outcome variable. Variables with *p* < 0.1 in univariate analysis were entered into multivariable logistic regression models. Odds ratios (ORs) and 95% confidence intervals (CIs) were calculated. Continuous variables were analyzed as linear predictors. HIF-T (%) and HIF-T grade were analyzed separately as measures of staining extent and staining intensity, respectively.

In the tables and statistical analyses, *N* represents the total number of patients included in the study, whereas *n* represents the number of patients within each subgroup.

All statistical tests were two-sided, and a *p* value < 0.05 was considered statistically significant.

## 3. Results

### 3.1. Patient Characteristics

A total of 55 patients with histologically confirmed ESCC who completed neoadjuvant CCRT followed by surgical resection were included in the analysis. The median age was 58 years (range, 47–72), and the cohort was predominantly male (90.9%).

Before neoadjuvant treatment, 78.2% of patients were clinically staged as stage III disease. After surgery, pathological staging revealed 29 patients (52.7%) with stage I–II disease and 26 patients (47.3%) with stage III disease. Lymphovascular invasion was identified in 49.1% of cases, and perineural invasion in 29.1%. Baseline demographic and clinicopathologic characteristics are summarized in Table 1.

### 3.2. Tumor HIF-1α Expression According to Pathological Stage

Across the entire cohort, the median tumor HIF-1α expression (HIF-T%) was 29.4% (IQR, 10–55%). When stratified by pathological stage, a clear separation in expression levels emerged.

Patients with pathological stage III disease demonstrated significantly higher HIF-T% compared with those with stage I–II disease (median 40% [IQR, 13.8–55%] vs. 15% [IQR, 3.5–40%], *p* = 0.023) (Table 2). Notably, the distribution of staining intensity also shifted toward higher grades in advanced-stage tumors. Grade 2 expression was more frequent in stage III patients (11.5%) than in stage I–II patients (6.9%), while grade 0 expression was less common in stage III tumors (3.8% vs. 20.7%).

The distribution of HIF-T (%) according to pathological stage is additionally illustrated in Supplementary Figure S1.

### 3.3. Stromal HIF-1α Expression

Median stromal/non-tumor HIF-1α expression (HIF-N%) across all patients was 29.5% (IQR, 15–46.3%). Although stage III patients exhibited numerically higher HIF-N% than stage I–II patients (35% vs. 25%), this difference was not statistically significant (*p* = 0.175). Intensity grading also did not significantly differ between groups.

### 3.4. HIF-1α Expression According to Pathological T Stage

When analyzed by pathological T classification (Table 3), tumors classified as T2–3 demonstrated higher median HIF-T% than T1 tumors (30% [IQR, 10–55%] vs. 10% [IQR, 8–21.3%]), although this did not reach statistical significance (*p* = 0.072). Staining intensity distributions were comparable (*p* = 0.826), and stromal HIF-N% also showed no significant differences (*p* = 0.576).

### 3.5. HIF-1α Expression According to Pathological N Stage

A similar pattern was observed when patients were stratified by nodal status (Table 4). Patients with N2–3 disease had higher median HIF-T% compared with those with N0–1 disease (40% [IQR, 20–60%] vs. 20% [IQR, 6.3–50%]), although this difference was not statistically significant (*p* = 0.134). No significant differences were observed in staining grade distribution (*p* = 1.000) or stromal HIF-N% (*p* = 0.882).

### 3.6. Multivariable Analysis

To further evaluate whether HIF-1α expression was independently associated with advanced pathological stage, logistic regression analysis was performed using pathological stage III disease as the outcome variable (Table 5).

In univariate analysis, higher tumor HIF-T (%) was significantly associated with pathological stage III disease (OR 1.04, 95% CI 1.01–1.07, *p* = 0.023). HIF-T grade and angiolymphatic invasion (ALI) demonstrated borderline significance.

Variables with *p* < 0.1 in univariate analysis were subsequently entered into the multivariable model. In multivariable logistic regression analysis, higher HIF-T (%) remained independently associated with advanced pathological stage (OR 1.04, 95% CI 1.01–1.08, *p* = 0.031), whereas HIF-T grade and ALI were not statistically significant.

Stage III patients demonstrated substantially higher median HIF-T% levels than stage I–II patients, consistent with the observed association between HIF-1α expression and advanced pathological stage.

### 3.7. Exploratory Survival Analysis

Exploratory survival analysis was additionally performed to evaluate the association between HIF-1α expression and overall survival (OS). In Cox proportional hazards regression analysis, neither HIF-T (%) nor HIF-T grade demonstrated statistically significant associations with OS. These findings should be interpreted cautiously given the limited sample size and the potential influence of heterogeneous post-recurrence salvage treatments on long-term outcomes.

## 4. Discussion

This study shows that elevated tumor HIF-1α expression is associated with advanced pathological stage in ESCC patients treated with neoadjuvant CCRT. Notably, the strongest association was observed at the level of overall pathological staging rather than individual T or N classifications. These findings suggest that HIF-1α-mediated hypoxia signaling may reflect the cumulative biological aggressiveness of residual disease following multimodality treatment.

From a clinical perspective, pathological stage after neoadjuvant therapy remains one of the most robust prognostic determinants in ESCC [3,4]. However, conventional TNM classification does not fully capture the biological heterogeneity underlying treatment response [3,4]. Tumors with similar anatomical stage may exhibit markedly different sensitivities to chemoradiotherapy due to intrinsic molecular characteristics. Our findings indicate that increased tumor HIF-1α expression may represent a hypoxia-driven phenotype associated with incomplete tumor eradication after CCRT. This raises the possibility that HIF-1α could serve as a complementary biomarker to identify patients at higher risk of residual advanced disease despite standard therapy.

Several previous studies have also reported clinical associations between HIF-1α expression and aggressive disease characteristics in ESCC [13,14,15,16]. Matsuyama et al. demonstrated that elevated HIF-1α levels were associated with advanced pathological features [10]. Similarly, Ogawa et al. reported that high HIF-1α expression was associated with inferior outcomes in patients treated with chemoradiotherapy [11]. Zhao et al. further reported that HIF-1α overexpression was linked to advanced T stage as well as poorer disease-free survival in ESCC patients [5]. Sohda et al. reported that HIF-1α expression, particularly when combined with p53 and p21 status, predicted resistance to chemoradiotherapy in ESCC [12]. Other investigations have also suggested that hypoxia-related biomarkers may predict resistance to radiotherapy-based treatment strategies [17,18,19,20].

Compared with prior investigations emphasizing survival endpoints or composite biomarker panels, our study specifically evaluated quantitative tumor HIF-1α expression in relation to post-neoadjuvant pathological stage following standardized CCRT. Whereas earlier studies primarily emphasized survival endpoints or composite biomarker panels [10,11,12], we specifically examined the association between hypoxia signaling and residual pathological burden following standardized CCRT. Nevertheless, our sample size remains smaller than that of some previous cohorts, which may partially explain why associations with individual T and N classifications did not reach statistical significance. Although statistical significance was not reached in certain subgroup analyses, consistent directional increases in HIF-T (%) were observed across more advanced pathological categories, suggesting that limited statistical power may have contributed to these nonsignificant findings.

A notable finding of this study was that tumor HIF-1α expression demonstrated a stronger association with overall pathological stage than with individual T or N classifications. This pattern may reflect underlying biological heterogeneity or the complex interplay between local tumor invasion and lymphatic dissemination. It is also plausible that overall pathological stage integrates multiple dimensions of tumor behavior, thereby amplifying detectable associations compared with isolated T or N components. Similar observations have been reported in other studies investigating hypoxia-related biomarkers in gastrointestinal malignancies [17,21].

From a translational perspective, hypoxia-related biomarkers such as HIF-1α may provide additional value in refining risk stratification in ESCC. Identification of tumors with high hypoxia signaling may help recognize patients who are less likely to achieve favorable pathological responses after neoadjuvant therapy. Emerging therapeutic strategies targeting tumor hypoxia or HIF signaling pathways have also been proposed [21,22,23], including hypoxia-activated prodrugs and agents that inhibit HIF-mediated transcriptional activity [13,14,15]. In addition, advances in molecular profiling and functional imaging techniques may allow more accurate assessment of tumor hypoxia in vivo. Integrating immunohistochemical markers such as HIF-1α with molecular or imaging-based biomarkers may therefore improve patient selection and guide individualized treatment strategies in the future.

However, not all studies have demonstrated consistent prognostic effects of HIF-1α. Zhang highlighted variability in hypoxia marker performance across different tumor contexts [24]. Rashid et al. further described bidirectional regulation of HIF-1α depending on microenvironmental and genetic factors [25], while Schöning et al. emphasized the distinct yet overlapping roles of HIF-1α and HIF-2α in treatment resistance and cancer stem cell maintenance [26]. Differences in antibody selection, scoring methodology, cutoff definitions, patient ethnicity, and treatment protocols likely contribute to inter-study heterogeneity. These factors underscore the importance of standardized evaluation when interpreting hypoxia biomarkers.

Interestingly, although both the proportion of HIF-1α-positive tumor cells (HIF-T%) and staining intensity were evaluated in this study, only HIF-T% remained independently associated with advanced pathological stage in multivariable analysis. This observation may provide additional biological insight into the role of tumor hypoxia in residual ESCC following neoadjuvant chemoradiotherapy. While these two parameters represent distinct dimensions of HIF-1α expression—namely spatial extent versus cellular intensity—they may be partially correlated in practice. Our findings suggest that the extent of hypoxia distributed throughout the tumor mass may be more relevant to residual pathological burden than staining intensity alone.

From a mechanistic perspective, this finding is biologically plausible. Under hypoxic conditions, stabilization of HIF-1α activates transcriptional pathways involved in angiogenesis, metabolic adaptation, epithelial–mesenchymal transition, and treatment resistance [27]. When a larger proportion of tumor cells is exposed to hypoxia, a broader fraction of the tumor population may simultaneously engage these adaptive pathways, thereby promoting resistance to chemoradiotherapy and persistent tumor viability. In contrast, staining intensity reflects the magnitude of HIF-1α expression at the individual cellular level, which may be influenced by transient microenvironmental fluctuations or technical variability, and may therefore incompletely represent the overall hypoxic burden within the tumor. Hypoxia is also known to contribute to radioresistance by reducing oxygen-mediated fixation of radiation-induced DNA damage and to chemoresistance through modulation of DNA damage response pathways and cellular metabolic reprogramming [23]. These biological effects are likely to exert greater impact when hypoxia is spatially widespread rather than confined to limited tumor regions.

Importantly, HIF-1α expression in the present study was assessed using post-CCRT surgical specimens. Therefore, the observed expression patterns may reflect not only intrinsic tumor hypoxia present before treatment, but also adaptive responses induced by chemoradiotherapy. This distinction is clinically relevant because intrinsic hypoxia may function as a predictive biomarker of treatment resistance, whereas treatment-induced HIF-1α upregulation may instead represent a biological characteristic of residual tumor cells that survived therapeutic stress [28,29]. Accordingly, the present findings should be interpreted as reflecting the biological features of residual disease rather than definitive predictive value. Nevertheless, given the retrospective design and the semi-quantitative nature of immunohistochemical assessment, these findings should be interpreted cautiously, and further studies incorporating pre-treatment evaluation and quantitative hypoxia assessment are warranted.

Several limitations should be acknowledged. First, the retrospective and single-institution design introduces inherent selection bias and may limit the generalizability of the findings to other populations or treatment settings. Second, the relatively small sample size (n = 55) limits statistical power, particularly for subgroup analyses of T and N categories. In addition, variability in baseline clinical stage was limited within the cohort, as the majority of patients were clinically stage III at diagnosis. This reflects the typical selection criteria for neoadjuvant chemoradiotherapy followed by surgery in ESCC, whereas early-stage disease is often managed with upfront surgery and metastatic disease is generally not considered resectable. Third, although exploratory overall survival analysis was additionally performed, no statistically significant association between HIF-1α expression and long-term clinical outcomes was observed in the present cohort. Importantly, survival and recurrence patterns may be influenced by subsequent salvage therapies, making it difficult to isolate the independent prognostic contribution of HIF-1α expression on overall prognosis. Furthermore, because immunohistochemical evaluation was performed on post-treatment surgical specimens, neoadjuvant therapy itself may have influenced HIF-1α expression levels. Finally, although immunohistochemical evaluation is clinically practical, it does not fully provide mechanistic insights at the transcriptomic or proteomic level. In addition, standardized quantitative approaches such as H-score assessment, interobserver reproducibility analysis, and digital image analysis were not available in the present retrospective cohort and may help improve biomarker standardization in future studies.

Future research should further clarify the clinical utility of hypoxia-related biomarkers in ESCC. Although tumor HIF-1α expression was associated with advanced pathological stage after neoadjuvant CCRT, its role in guiding treatment decisions remains to be established. Prospective studies with larger cohorts are warranted to determine whether hypoxia-driven biomarkers can guide individualized treatment and improve clinical outcomes [13,14,23,24,25].

## 5. Conclusions

In this cohort of ESCC patients treated with neoadjuvant CCRT followed by surgery, elevated tumor HIF-1α expression was associated with advanced pathological stage at resection. These findings support a potential role of hypoxia-related signaling in treatment-resistant disease. Tumor HIF-1α expression may serve as a biologically relevant biomarker for identifying patients less likely to respond to neoadjuvant therapy. Further validation in larger prospective cohorts is warranted.

## Figures and Tables

**Table 1:** Clinicopathologic characteristics of the study cohort (*N* = 55).

Characteristics	Number
Gender	
Female	5 (9.1%)
Male	50 (90.9%)
Age	58 (47–72)
Histology	
SCC	55 (100%)
Pre-neoadjuvant clinical staging	
I	1 (1.8%)
II	11 (20.0%)
III	43 (78.2%)
Pathologic Staging	
I	4 (7.2%)
II	25 (45.5%)
III	26 (47.3%)
Lymphovascular invasion	27 (49.1%)
Peri-neural invasion	16 (29.1%)

Pre-neoadjuvant clinical staging was determined according to the 8th edition AJCC TNM classification system. Pathological staging was assigned based on postoperative histopathologic evaluation according to the same AJCC criteria. Abbreviations: SCC, squamous cell carcinoma; *N*, total number of patients.

**Table 2:** Comparison of HIF-1α expression according to pathological stage.

	I–II (*n* = 29)	III (*n* = 26)	*p* Value
HIF-T (%), median (IQR)	15 (3.5–40)	40 (13.8–55)	0.023 *
HIF-T, *n* (%)			0.164
Grade 0	6 (20.7%)	1 (3.8%)	
Grade 1	21 (72.4%)	22 (84.6%)	
Grade 2	2 (6.9%)	3 (11.5%)	
HIF-N (%), median (IQR)	25 (15–37.5)	35 (18.8–46.3)	0.175
HIF-N, *n* (%)			0.233
Grade 0	3 (10.3%)	0 (0%)	
Grade 1	23 (79.3%)	25 (96.2%)	
Grade 2	3 (10.3%)	1 (3.8%)	

Statistical analysis was performed using the Mann–Whitney U test, chi-square test, or Fisher’s exact test as appropriate. * *p* < 0.05. Abbreviations: HIF-T, HIF-1α expression in tumor cells; HIF-N, HIF-1α expression in non-tumor (stromal) cells; *n*, number of patients in each subgroup.

**Table 3:** Comparison of HIF-1α expression according to pathological T stage (*n* = 55).

	T1 (*n* = 10)	T2–3 (*n* = 45)	*p* Value
HIF-T (%), median (IQR)	10 (8–21.3)	30 (10–55)	0.072
HIF-T, *n* (%)			0.164
Grade 0	1 (10.0%)	6 (13.3%)	
Grade 1	9 (90.0%)	34 (75.6%)	
Grade 2	0 (0%)	5 (11.1%)	
HIF-N (%), median (IQR)	25 (16.3–35)	30 (15–42.5)	0.576
HIF-N, *n* (%)			0.143
Grade 0	1 (10.0%)	2 (4.4%)	
Grade 1	7 (70.0%)	41 (91.1%)	
Grade 2	2 (20.0%)	2 (4.4%)	

Statistical analysis was performed using the Mann–Whitney U test, chi-square test, or Fisher’s exact test as appropriate. Abbreviations: HIF-T, HIF-1α expression in tumor cells; HIF-N, HIF-1α expression in non-tumor (stromal) cells; *n*, number of patients in each subgroup.

**Table 4:** Comparison of HIF-1α expression according to pathological N stages (*n* = 55).

	N0–1 (*n* = 44)	N2–3 (*n* = 11)	*p* Value
HIF-T (%), median (IQR)	20 (6.3–50)	40 (20–60)	0.134
HIF-T, *n* (%)			1.000
Grade 0	6 (13.6%)	1 (9.1%)	
Grade 1	34 (77.3%)	9 (81.8%)	
Grade 2	4 (9.1%)	1 (9.1%)	
HIF-N (%), median (IQR)	25 (15–40)	35 (15–40)	0.882
HIF-N, *n* (%)			0.781
Grade 0	3 (6.8%)	0 (0%)	
Grade 1	37 (84.1%)	11 (100%)	
Grade 2	4 (9.1%)	0 (0%)	

Statistical analysis was performed using the Mann–Whitney U test, chi-square test, or Fisher’s exact test as appropriate. Abbreviations: HIF-T, HIF-1α expression in tumor cells; HIF-N, HIF-1α expression in non-tumor (stromal) cells; *n*, number of patients in each subgroup.

**Table 5:** Logistic regression analysis of factors associated with advanced pathological stage (pStage III).

Variable	Univariate OR	95% CI	*p* Value	Multivariable OR	95% CI	*p* Value
Age	1.01	0.97–1.05	0.61			
Sex (Male vs. Female)	1.42	0.28–7.10	0.67			
HIF-T (%)	1.04	1.01–1.07	0.023	1.04	1.01–1.08	0.031 *
HIF-T grade	1.75	0.93–3.30	0.08	1.21	0.61–2.40	0.58
HIF-N (%)	1.01	0.99–1.03	0.22			
HIF-N grade	1.10	0.70–1.73	0.68			
ALI	2.05	0.97–4.32	0.06	1.63	0.74–3.62	0.23
PNI	1.62	0.78–3.38	0.19			

Univariate and multivariable logistic regression analyses were performed to identify factors associated with advanced pathological stage (pStage III). Odds ratios (ORs) with 95% confidence intervals (CIs) are presented. * *p* < 0.05. Abbreviations: OR, odds ratio; CI, confidence interval; HIF-T expression in tumor cells; HIF-N expression in non-tumor stromal cells; ALI, angiolymphatic invasion; PNI, perineural invasion.

## Data Availability

The original contributions presented in this study are included in the article and Supplementary Material. Further inquiries can be directed to the corresponding author.

## Supplementary Materials

The following supporting information can be downloaded at https://www.mdpi.com/article/10.3390/cimb48050525/s1.

## Abbreviations

The following abbreviations are used in this manuscript:

EC	Esophageal cancer
ESCC	Esophageal squamous cell carcinoma
CCRT	Concurrent chemoradiotherapy
HIF-1α	Hypoxia-inducible factor-1 alpha
VEGF	Vascular endothelial growth factor
TNM	Tumor-node-metastasis
AJCC	American Joint Committee on Cancer
FFPE	Formalin-fixed paraffin-embedded
IRB	Institutional Review Board
VMAT	Volumetric modulated arc therapy
SAD	Source-to-axis distance
5-FU	5-fluorouracil
IHC	Immunohistochemistry
H&E	Hematoxylin and eosin
PBS	Phosphate-buffered saline
HRP	Horseradish peroxidase
DAB	3,3′-diaminobenzidine
SPSS	Statistical Package for the Social Sciences
IQR	Interquartile range
OR	Odds ratio
CI	Confidence intervals
ALI	Angiolymphatic invasion
PNI	Perineural invasion
