# Supplementary material for: Elevated Tumor HIF-1α Expression Correlates with Advanced Pathological Stage Following Neoadjuvant Concurrent Chemoradiotherapy in Esophageal Squamous Cell Carcinoma
Source: Curr Issues Mol Biol. 2026 May 18;48(5):525. doi: 10.3390/cimb48050525 (PMC13204692; doi:10.3390/cimb48050525)

Supplementary Figure S1. Distribution of HIF-T (%) according to pathological stage. Boxplots show the distribution and median values of HIF-T (%) in patients with pathological stage I–II and stage III disease.

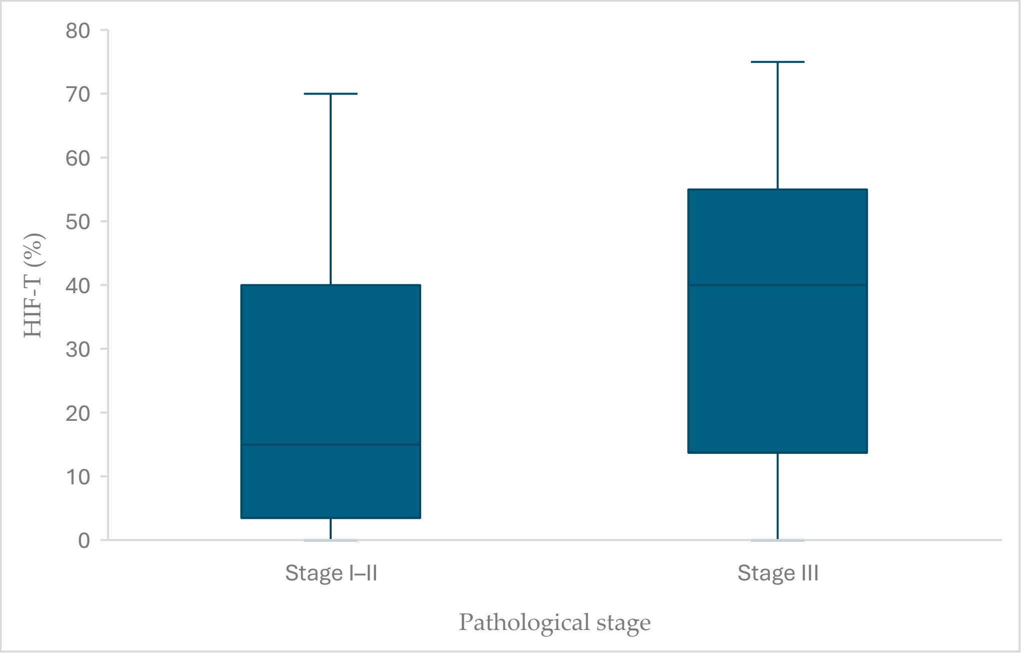

Supplement: Supplementary file 1 [file cimb-48-00525-s001.zip › cimb-4306832-supplementary.pdf]
